# Examination of the efficacy of acute L-alanyl-L-glutamine ingestion during hydration stress in endurance exercise

**DOI:** 10.1186/1550-2783-7-8

**Published:** 2010-02-03

**Authors:** Jay R Hoffman, Nicholas A Ratamess, Jie Kang, Stephanie L Rashti, Neil Kelly, Adam M Gonzalez, Michael Stec, Steven Anderson, Brooke L Bailey, Linda M Yamamoto, Lindsay L Hom, Brian R Kupchak, Avery D Faigenbaum, Carl M Maresh

**Affiliations:** 1 The College of New Jersey, Department of Health and Exercise Science, Ewing NJ 08628, USA; 2 University of Connecticut, Department of Kinesiology, Storrs CT 06269, USA

## Abstract

**Background:**

The effect of acute L-alanyl-L-glutamine (AG; Sustamine™) ingestion on performance changes and markers of fluid regulation, immune, inflammatory, oxidative stress, and recovery was examined in response to exhaustive endurance exercise, during and in the absence of dehydration.

**Methods:**

Ten physically active males (20.8 ± 0.6 y; 176.8 ± 7.2 cm; 77.4 ± 10.5 kg; 12.3 ± 4.6% body fat) volunteered to participate in this study. During the first visit (T1) subjects reported to the laboratory in a euhydrated state to provide a baseline (BL) blood draw and perform a maximal exercise test. In the four subsequent randomly ordered trials, subjects dehydrated to -2.5% of their baseline body mass. For T2, subjects achieved their goal weight and were not rehydrated. During T3 - T5, subjects reached their goal weight and then rehydrated to 1.5% of their baseline body mass by drinking either water (T3) or two different doses (T4 and T5) of the AG supplement (0.05 g·kg^-1 ^and 0.2 g·kg^-1^, respectively). Subjects then exercised at a workload that elicited 75% of their VO_2 _max on a cycle ergometer. During T2 - T5 blood draws occurred once goal body mass was achieved (DHY), immediately prior to the exercise stress (RHY), and immediately following the exercise protocol (IP). Resting 24 hour (24P) blood samples were also obtained. Blood samples were analyzed for glutamine, potassium, sodium, aldosterone, arginine vasopressin (AVP), C-reactive protein (CRP), interleukin-6 (IL-6), malondialdehyde (MDA), testosterone, cortisol, ACTH, growth hormone and creatine kinase. Statistical evaluation of performance, hormonal and biochemical changes was accomplished using a repeated measures analysis of variance.

**Results:**

Glutamine concentrations for T5 were significantly higher at RHY and IP than T2 - T4. When examining performance changes (difference between T2 - T5 and T1), significantly greater times to exhaustion occurred during T4 (130.2 ± 340.2 sec) and T5 (157.4 ± 263.1 sec) compared to T2 (455.6 ± 245.0 sec). Plasma sodium concentrations were greater (p < 0.05) at RHY and IP for T2 than all other trials. Aldosterone concentrations at RHY and IP were significantly lower than that at BL and DHY. AVP was significantly elevated at DHY, RHY and IP compared to BL measures. No significant differences were observed between trials in CRP, IL-6, MDA, or in any of the other hormonal or biochemical measures.

**Conclusion:**

Results demonstrate that AG supplementation provided a significant ergogenic benefit by increasing time to exhaustion during a mild hydration stress. This ergogenic effect was likely mediated by an enhanced fluid and electrolyte uptake.

## Background

During dehydration fluid moves from the plasma to both intracellular and extracellular spaces and then eventually back to the circulation [1,2]. Pressure changes involving hydrostatic, oncotic and osmotic forces control the dynamics of fluid movement [1]. This has important implications for thermoregulation and athletic performance. Significant performance decrements have been shown with hypohydration levels of only 2% [3]. Considering that a thirst sensation may not develop until this level of hypohydration has already been reached, it becomes critical for athletes to rehydrate before they feel the need to drink.

Several sport drinks are marketed to be a more effective means of promoting rehydration and maintaining exercise performance than water alone. However, little research is available to support the efficacy of these drinks during relatively short duration endurance exercise (≤ 2 hr). Water appears as effective as any sports drink during exercise in maintaining performance and thermoregulation [4]. Interestingly, recent advances in sport supplements suggest the use of certain organic osmolytes such as glycine betaine may provide some protection of intracellular fluid volume [5]; however, its ability to affect performance is not clear.

Recent research demonstrates that certain amino acids may also promote rehydration by enhancing water and electrolyte absorption [6]. Glutamine has been reported to increase electrolyte and water absorption in both animal and human subjects suffering from intestinal infections [7-9], but not in others [10]. However, differences may be related to the stability issues related to glutamine. Fürst [11] suggested that glutamine derivatives such as alanyl-glutamine may be more stable than glutamine by itself, especially at low pH. This could be a potential scenario during exercise when increases in lactic acid are common. Lima and colleagues [6] reported that alanine and glutamine together is more stable than glutamine alone in increasing electrolyte and water absorption, likely via an improvement in ion transporters within intestinal epithelia.

Both glutamine and alanine/glutamine in combination have been shown to be effective for antioxidant defense during situations of severe illness [12-14]. In addition, glutamine has been shown to be an effective modulator of the immune response to exercise [15] and possibly improve athletic performance [16]. However, there is considerable debate in this area [17], which justifies further investigation. Thus, the purpose of this study was to examine the efficacy of two different doses (0.2 g·kg^-1 ^and 0.05 g·kg^-1^) of the dipeptide L-Alanyl-L-Glutamine on performance, recovery and the fluid regulatory response during an exhaustive endurance exercise protocol following a 2.5% dehydration stress. In addition, the effect of this dipeptide L-Alanyl-L-Glutamine on endocrine and biochemical markers of inflammation, oxidative stress and immune response during the exercise and dehydration stress was also examined.

## Methods

### Subjects

Ten college-aged males (20.8 ± 0.6 y; 176.8 ± 7.2 cm; 77.4 ± 10.5 kg; 12.3 ± 4.6% body fat) volunteered for this study. Prior to participation, each subject was informed of all procedures, risks and benefits and completed written informed consent approved by the Institutional Review Board. Subjects ceased use of additional nutritional supplements for at least four weeks prior to the study. Screening for supplement use was accomplished via a health history questionnaire completed during the subject recruitment phase.

### Protocol

Prior to the onset of the study subjects reported to the Human Performance Laboratory (HPL) for determination of baseline body mass. These measures occurred on nonconsecutive days approximately one week before the start of experimental testing. Subjects were weighed during these visits in a postabsorptive, euhydrated state to establish a baseline body weight. Upon arrival, subjects voided their bladder for urinary measures of osmolality (U_osm_) by freezing point depression (Model 3320; Micro-Sample Osmometer, Advanced Instruments, Inc., Norwood, MA) and urine specific gravity (U_sg_) by refractometry (A300CL-E01, Atago, Tokyo, Japan) to document euhydration on all preliminary days; U_sg _≤ 1.020 was defined as euhydration [18]. During the first session (T1) subjects performed a graded maximal aerobic capacity test () on an electromagnetically braked cycle ergometer (Ergo 800, SensorMedics, Inc., Yorba Linda, CA). The  test was administered to establish workloads for the subsequent endurance tests. Oxygen consumption (), respiratory exchange ratio (RER), and minute ventilation () were measured (ULTIMA, MedGraphics Corporation, St. Paul, MN). Gas analyzers were calibrated using gases provided by MedGraphics Corporation: 1) calibration gas: 5% CO_2_, 12% O_2_, balance N_2_; and 2) reference gas: 21% O_2_, balance N_2_. Gas calibration was conducted before each trial. Heart rate (HR) was measured via telemetry (Pacer, Polar CIC, Inc., Port Washington, NY).

On four subsequent visits (T2-T5) to the HPL, subjects dehydrated by 2.5% of baseline body mass. On the occasion that a dehydration protocol was not employed the subjects reported to the HPL in a euhydrated state to provide a baseline blood draw and perform the exercise protocol. This trial (T1) provided baseline performance data in optimal conditions without a hydration stress. All performance comparisons were made to this trial. In one trial (T2) subjects achieved their goal weight and rested in a recumbent position for 45 minutes before commencing the exercise session. In the subsequent three trials subjects reached their goal weight and then rehydrated to -1.5% of their baseline body mass by drinking either water (T3) or two different doses (T4 and T5) of the alanine-glutamine (AG) supplement (0.05 g·kg^-1 ^and 0.2 g·kg^-1^, respectively). During the hydration trials (T3 - T5), the exercise protocol began 45 minutes following rehydration. The order of the trials was randomized

### Dehydration Protocol

On the night before testing (1700 hrs) subjects reported to the HPL for weight and U_sg _measures to verify euhydration. Subjects were instructed to not consume any food or water until the next day when they reported back to the HPL (0700 hrs). This resulted in an average body mass change of -1.03 ± 1.3%. On the morning of trials T2 - T5 subjects reported to the HPL were weighed and then began the active dehydration protocol to achieve the desired weight loss. The active dehydration protocol consisted of walking on a motorized treadmill at 3.4 mi·h^-1 ^at a 2% incline. Subjects were fully clothed in a training suit (long cotton heavy weight fleece sweat pants and top). Nude body weight, HR, and rating of perceived exertion were monitored at 20-minute increments. The subjects continued to walk until they (a) lost 2.5% of their body mass, (b) met preset safety criteria, (c) displayed signs or symptoms of an exercise-induced heat illness, or (d) reached volitional fatigue. Dehydration was verified via U_sg_, U_osm _and plasma osmolality (P_osm_). Total exercise time to achieve hypohydration (-2.5% weight loss) was 62.5 ± 44.2 min. There were no significant differences in time to reach goal body mass among trials.

### Supplement Schedule

Subjects rehydrated to -1.5% body mass by consuming the supplement or the placebo (water) following the dehydration protocol. The L-alanyl-L-glutamine supplement (0.2 g·kg^-1 ^or 0.05 g·kg^-1 ^body mass per liter) marketed as "*Sustamine*™" (Kyowa Hakko USA, New York, NY) was mixed with water and was indistinguishable in appearance and taste from the placebo.

### Time to Exhaustion Test

After the dehydration and rehydration phase, subjects began the exercise protocol. Subjects exercised at a workload that elicited 75% of their  on a cycle ergometer. Subjects were encouraged to give their best effort during each trial, and were verbally encouraged throughout each exercise trial. , RER, , RER, and HR, were measured continuously. HR and blood pressure (BP) were recorded before and at the conclusion of exercise. Time to exhaustion was determined as the time that the subject could no longer maintain the workload and/or reached volitional exhaustion.

### Blood Measures

A baseline (BL) blood draw occurred during T1. No other blood was drawn during that trial. The BL blood sample was drawn following a 15-min equilibration period prior to exercise. All day of trial blood samples (DHY, RHY and IP) were obtained using a 20-gauge Teflon cannula placed in a superficial forearm vein using a 3-way stopcock with a male luer lock adapter. The cannula was maintained patent using an isotonic saline solution (with 10% heparin). During trials T2 - T5 blood draws occurred once goal body mass was achieved (DHY), immediately prior to the exercise stress (RHY) and immediately following the exercise protocol (IP). IP blood samples were taken within 15 seconds of exercise cessation. Subjects returned to the laboratory 24-h post-exercise for an additional blood draw (24P). All BL and 24P blood samples were drawn with a plastic syringe while the subject was in a seated position. These blood samples were obtained from an antecubital arm vein using a 20-gauge disposable needle equipped with a Vacutainer^® ^tube holder (Becton Dickinson, Franklin Lakes, NJ) with the subject in a seated position. Each subjects' blood samples were obtained at the same time of day during each session. Blood samples were drawn into plain or EDTA treated tubes (Vacutainer, Becton Dickinson, Franklin Lakes, NJ). Blood samples were analyzed in triplicate for hematocrit via microcapillary technique and hemoglobin via the cyanmethemoglobin method (Sigma Diagnostics, St. Louis, MO). The remaining whole blood was centrifuged for 15 min at 1500 g at 4°C. Resulting plasma and serum were aliquoted and stored at -80°C until analysis. Samples were thawed only once.

### Biochemical and Hormonal Analyses

Serum testosterone (TEST), cortisol (CORT) and growth hormone (GH) concentrations were determined using enzyme immunoassays (EIA) and enzyme-linked immunosorbent assays (ELISA) (Diagnostic Systems Laboratory, Webster, TX). Serum aldosterone (ALD) and IL-6 concentrations were determined using an EIA assay (ALPCO Diagnostics, Salem, NH). Plasma arginine vasopressin (AVP) concentrations were determined using an EIA assay (Cayman Chemical Co, Ann Arbor, MI). Plasma adrenocorticotrophic hormone (ACTH, ALPCO Diagnostics, Salem, NH), C-reactive protein (CRP, Diagnostic Systems Laboratory, Webster, TX), and malondialdehyde (MDA, Cell Biolabs Inc., San Diego, CA) concentrations were assayed in duplicate via ELISA. Determination of serum immunoreactivity values was made using a SpectraMax340 Spectrophotometer (Molecular Devices, Sunnyvale, CA). Serum creatine kinase (CK) concentrations were determined at 340 nm on a spectrophotometer (Pointe Scientific, Inc, Canton, MI). Plasma glutamine, glucose and lactate (La^-^) concentrations were determined in duplicate with an automated analyzer (Analox GM7 enzymatic metabolite analyzer, Analox Instruments USA, Lunenburg, MA). Plasma sodium and potassium concentrations were assessed via ion-selective electrodes (EasyElectrolyte, Medica, Bedford, MA). To eliminate inter-assay variance, all samples were analyzed in the same assay run. Intra-assay variance for all assays was < 10%. Plasma volume shifts following the workout were calculated using the formula of Dill & Costill [19].

### Statistical Analysis

All data were assessed and met assumptions for normal distribution, homogeneity of variance, and sample independence. Statistical evaluation of performance, hormonal and biochemical changes were analyzed using a repeated measures analysis of variance (ANOVA). In the event of a significant F- ratio, LSD post-hoc tests were used for pairwise comparisons. Prior to the ANOVA, Plasma volume shifts, performance comparisons, and area under the curve (AUC) calculated using standard trapezoidal technique were analyzed using a One-Way ANOVA. Significance was accepted at an alpha level of *p *≤ *0.05*. All data are reported as mean ± SD.

## Results

**Figure 1:**
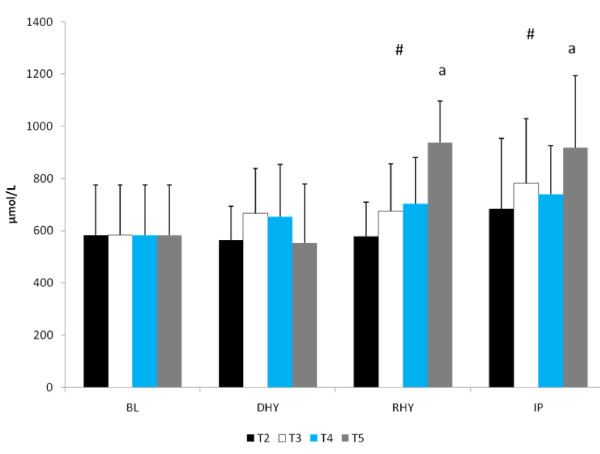
**Plasma Glutamine Concentrations. There was a significant main effect for trial between T2 and T5**. # = significant main effect for time versus BL and DHY; a = significantly different from T2, T3, and T4.

**Figure 2:**
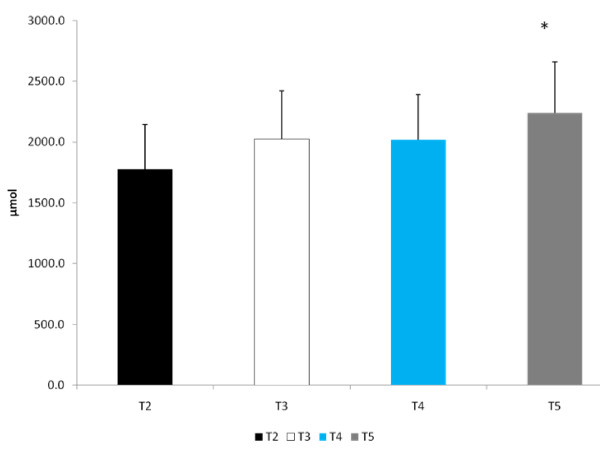
**AUC Glutamine**. * = Significantly different from T2

U_sg _(1.026 ± 0.004), U_osm _(813 ± 299 mOsm) and P_osm _(297.0 ± 4.6 mOsm) were similar for all trials at DHY. These results reflected the overnight fasting and exercise-induced dehydration performed during prior to each trial. Plasma glutamine concentrations were significantly higher for all groups at RHY and IP compared to BL (p = 0.002 and p = 0.000, respectively) and DHY (p = 0.001 and p = 0.000, respectively) (Figure 1). [Glutamine] for T5 were significantly higher at RHY and IP than T2 - T4. AUC analysis showed significantly greater [glutamine] for T5 at all time points compared to the other experimental trials (see Figure 2).

**Figure 3:**
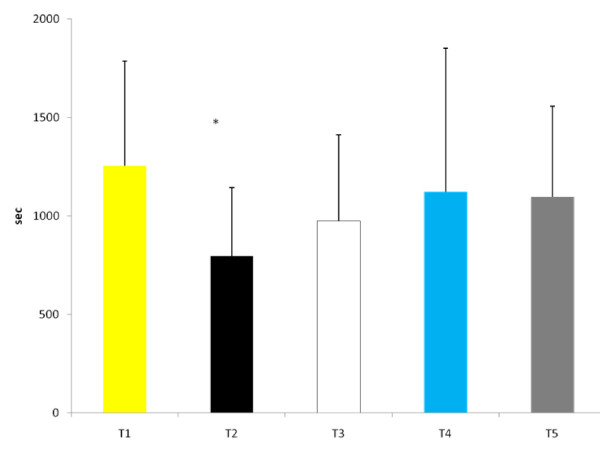
**Time to Exhaustion**. * Significantly different from all other trials.

**Figure 4:**
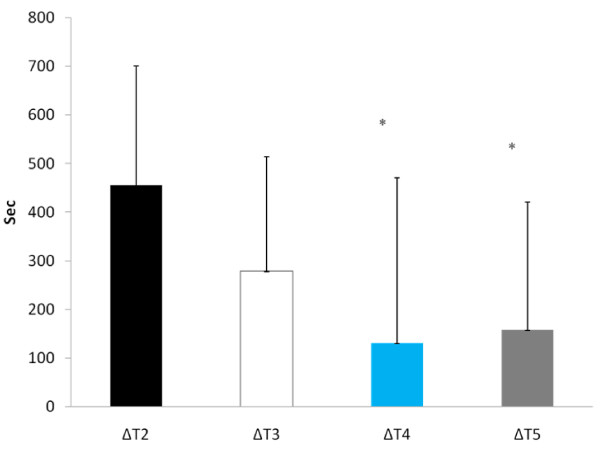
**Δ Time to Exhaustion**. * = Significantly different from ΔT2

Time to exhaustion was significantly reduced during T2 than at any other experimental trial (see Figure 3). When examining Δ performance (difference between each experimental trial and T1), time to exhaustion was significantly greater during T4 (130.2 ± 340.2 sec) and T5 (157.4 ± 263.1 sec) compared to T2 (455.6 ± 245.0 sec, p = 0.05 and p = 0.01, respectively) (Figure 4). No other between trial differences were noted. Cardiovascular changes during exercise are depicted in Table 1. No significant differences in either resting or post-exercise HR occurred between trials. In addition, no differences occurred in resting BP between trials, however, systolic BP post-exercise was significantly lower at T2 and T3 compared to T1. No other differences existed in systolic or diastolic BP response between trials. No changes in RER occurred between trials.

**Table 1:** Cardiovascular Changes during Exercise Protocol

Variable	T1	T2	T3	T4	T5
Resting Heart Rate (beats·min^-1^)	75.7 ± 14.6	78.6 ± 15.4	72.9 ± 13.8	76.7 ± 17.6	76.9 ± 15.8

IP Heart Rate (beats·min^-1^)	180.2 ± 13.8	187.8 ± 9.6	179.7 ± 18.0	183.0 ± 12.5	184.2 ± 13.0

Resting SBP (mmHg)	117.0 ± 6.0	112.4 ± 4.8	111.5 ± 5.5	114.8 ± 5.2	113.0 ± 7.7

IP SBP (mmHg)	167.3 ± 6.0	131.3 ± 8.1*	136.4 ± 20.3*	150.3 ± 23.0	152.5 ± 19.6

Resting DBP (mmHg)	77.3 ± 3.6	74.7 ± 4.8	75.4 ± 3.8	79.0 ± 2.7	77.2 ± 5.9

IP DBP (mmHg)	88.4 ± 7.0	86.0 ± 3.5	84.0 ± 9.4	88.3 ± 11.6	84.8 ± 11.9

RER	1.12 ± 0.09	1.10 ± 0.07	1.12 ± 0.07	1.08 ± 0.10	1.07 ± 0.08

IP = immediate post; SBP = systolic blood pressure; DBP = diastolic blood pressure. * = significant difference versus T1. All data are reported as mean ± SD.

There were significant main effects for both La^- ^(p = 0.000) and GLU (p = 0.000) responses to the exercise protocol (Table 2). There were also significant elevations at IP in both of these variables compared to all other time points. However, there were no significant differences between trials. A main effect for time (p = 0.011) also occurred for plasma osmolality. P_osm _at IP (300.4 ± 16.7 mOsm) was significantly elevated compared to BL (295.0 ± 3.9 mOsm, p = 0.010) and RHY (293.9 ± 4.9 mOsm, p = 0.002) but, not DHY (297.0 ± 4.5 mOsm, p = 0.100). No other significant differences were noted. In addition, no between trial differences in P_osm _were observed. A significant main effect for time (p = 0.001) was also observed for plasma potassium concentrations. Plasma potassium was significantly elevated at IP compared to BL (p = 000), DHY (p = 0.000) and RHY (p = 0.017). No other differences were noted and no between trial effects were observed. A significant main effect for time (p = 0.000) was also observed for plasma sodium. Plasma sodium concentrations at IP and DHY were significantly greater than that observed at BL (p = 0.000 and p = 0.000, respectively) and RHY (p = 0.000 and p = 0.000, respectively). When collapsed across time, plasma sodium concentrations were significantly greater at T2 than compared to all other experimental conditions. Plasma sodium concentrations were also significantly greater for T2 than all other experimental trials at RHY (p = 0.000) and IP (p = 0.000). AUC analysis also demonstrated a significantly greater sodium concentration for T2 compared to all other trials.

**Table 2:** Plasma Lactate, Glucose, Osmolality and Electrolyte Response to Exercise

Variable		T2	T3	T4	T5
				
	Time Point				
Lactate (mmol·L^-1^)	DHY	1.9 ± 0.6	1.9 ± 0.6	2.0 ± 0.6	1.7 ± 0.6
	
	RHY	1.8 ± 0.5	2.1 ± 0.4	2.0 ± 0.5	2.1 ± 0.4
	
	IP*	11.1 ± 2.3	11.9 ± 2.2	9.9 ± 4.2	11.7 ± 2.2

Glucose (mmol·L^-1^)	BL	5.8 ± 1.2	5.8 ± 1.2	5.8 ± 1.2	5.8 ± 1.2
	
	DHY	6.5 ± 1.8	6.4 ± 1.1	6.4 ± 1.4	5.7 ± 1.2
	
	RHY	5.9 ± 1.7	6.2 ± 1.1	6.4 ± 0.9	5.6 ± 1.2
	
	IP*	6.9 ± 1.6	8.6 ± 1.5	8.4 ± 1.9	7.4 ± 2.6

Osmolality (mOsm)	BL	295 ± 4	295 ± 4	295 ± 4	295 ± 4
	
	DHY	298 ± 5	298 ± 5	296 ± 4	298 ± 6
	
	RHY	298 ± 6	293 ± 5	292 ± 4	294 ± 4
	
	IP#	308 ± 5	299 ± 4	302 ± 5	303 ± 7

Potassium (mmol·L^-1^)	BL	4.1 ± 0.4	4.1 ± 0.4	4.1 ± 0.4	4.1 ± 0.4
	
	DHY	4.2 ± 0.9	4.0 ± 0.3	4.1 ± 0.3	4.0 ± 0.3
	
	RHY	4.1 ± 0.2	4.3 ± 0.3	4.3 ± 0.6	4.1 ± 0.4
	
	IP*	4.5 ± 0.7	4.5 ± 0.5	4.4 ± 0.4	4.5 ± 0.6

Sodium (mmol·L^-1^)	BL	139.4 ± 1.1	139.4 ± 1.1	139.4 ± 1.1	139.4 ± 1.1
	
	DHY*	141.7 ± 1.1	141.3 ± 1.6	141.1 ± 2.5	141.2 ± 1.4
	
	RHY	141.5 ± 1.5@	139.6 ± 1.9	138.7 ± 1.9	138.7 ± 1.6
	
	IP#	144.0 ± 2.2@	140.6 ± 1.8	140.7 ± 2.0	140.2 ± 1.3

* = Significant main effect compared to all other time points. # = Significant main effect compared to BL and RHY. @ = significantly different than T3 - T5. BL = baseline; DHY = dehydration; RHY = rehydration; IP = immediate post-exercise.

**Figure 5:**
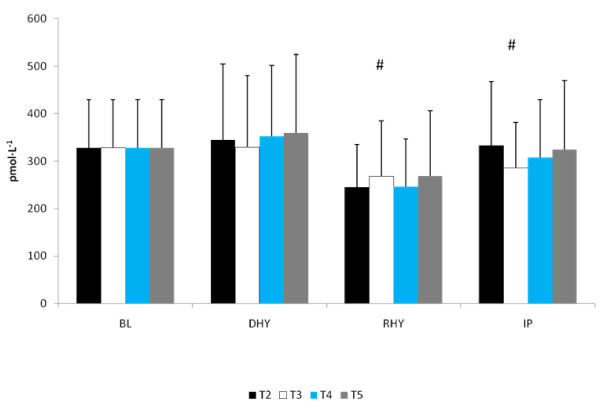
**Serum Aldosterone Response**. # = significant main effect for time between BL and DHY.

**Figure 6:**
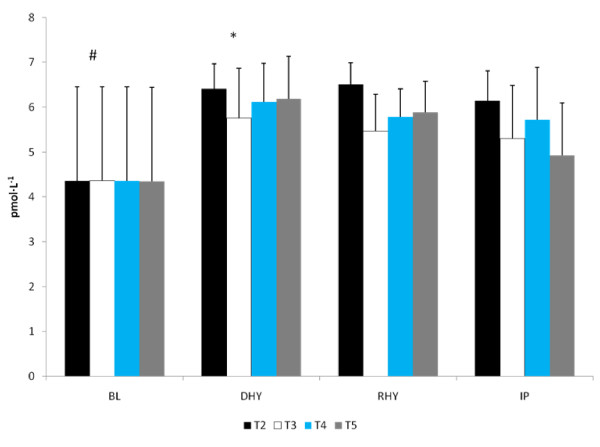
**Arginine Vasopressin**. # = significant main effect for time BL versus DHY, RHY and IP. * = Significant main effect between DHY and IP.

The ALD response to the experimental trials is presented in Figure 5. A significant main effect for time (p = 0.013) was observed. [ALD] at RHY and IP were significantly lower than that at BL and DHY (Figure 5). No other significant differences were noted and no significant interactions were observed. The plasma AVP responses are shown in Figure 6. A significant main effect for time (p = 0.000) was also observed. AVP was significantly elevated at DHY (p = 0.000), RHY (p = 0.000) and IP (p = 0.000) compared to BL measures. In addition, AVP concentrations at DHY were significantly higher (p = 0.05) than IP across all trials. There were no significant differences between trials, and no significant interactions between time and trial.

**Figure 7:**
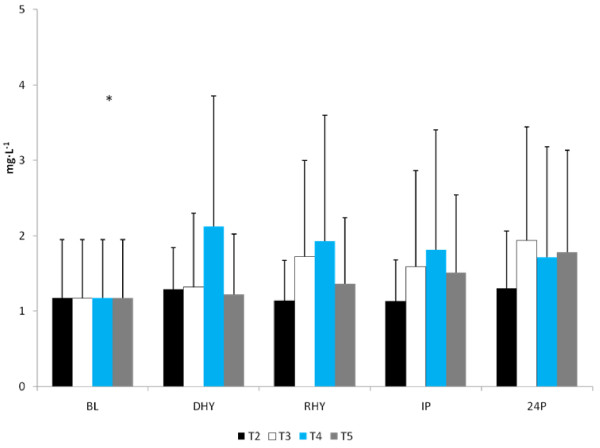
**C-Reactive Protein Response**. * = significant main effect for time BL.

**Figure 8:**
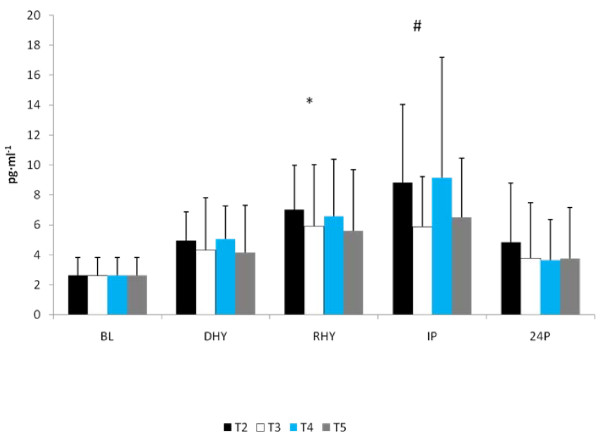
**IL-6 Response**. # = significant main effect for time versus BL, DHY and 24P; * significant main effect for time versus BL and 24P.

**Figure 9:**
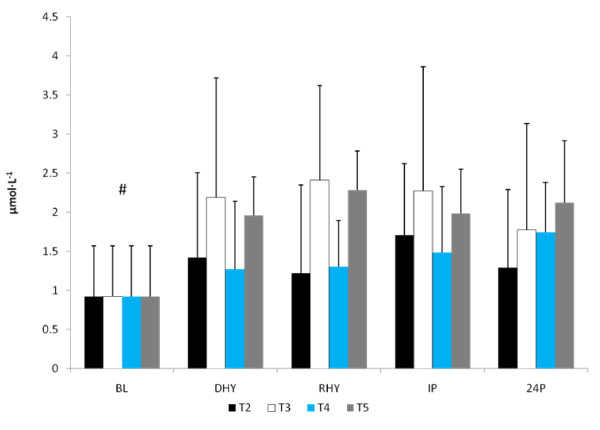
**MDA Response**. # = significant main effect for time versus DHY, RHY, IP, and 24P; There was a significant main effect for Trial between T3 and T5 versus T2 and T4.

No significant differences were observed between trials in CRP, IL-6, and MDA response to the exercise and hydration stress (see Figures 7, 8 and 9, respectively). A significant main effect for time was observed for both CRP (p = 0.000) and MDA (p = 0.000). BL concentrations for both of these variables were significantly lower than all other time points. There was a significant main effect for trial for MDA between T3 and T5 versus T2 (p = 0.004 and p = 0.008, respectively) and T4 (p = 0.05 and p = 0.011, respectively). Evaluation of the response of IL-6 revealed a significant main effect for time (p = 0.000). IL-6 concentrations were significantly greater at IP than at BL (p = 0.000), DHY (p = 0.000), and IP (p = 0.000). In addition, IL-6 concentrations at RHY were significantly higher than at BL (p = 0.000) and 24P (p = 0.006). AUC analysis for CRP, IL-6 and MDA did not reveal any significant differences between trials.

**Figure 10:**
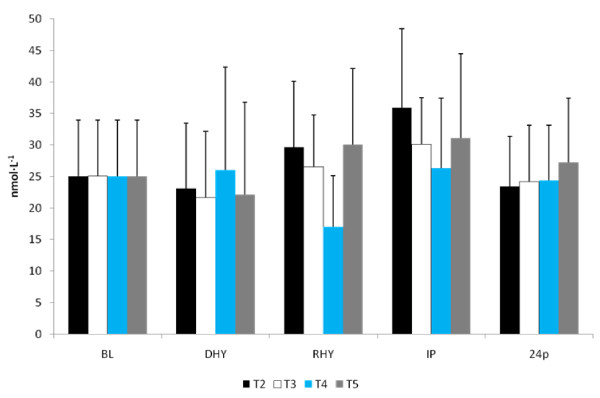
**Testosterone Response**.

**Figure 11:**
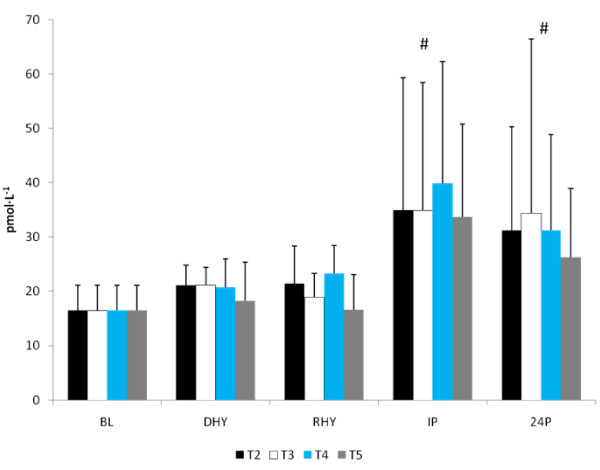
**ACTH Response**. # = significant main effect for time versus BL, DHY and RHY

**Figure 12:**
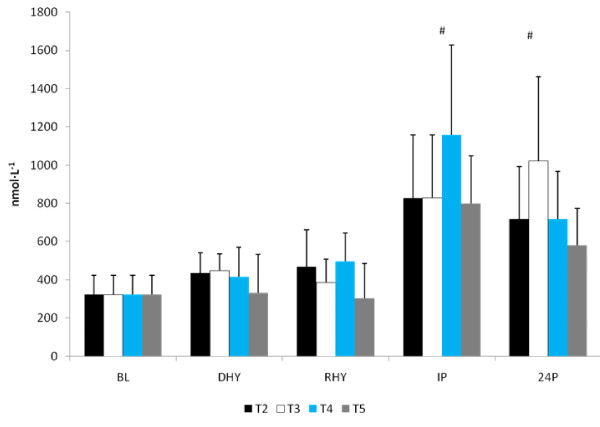
**Cortisol Response**. # = significant main effect for time versus BL, DHY and RHY

**Figure 13:**
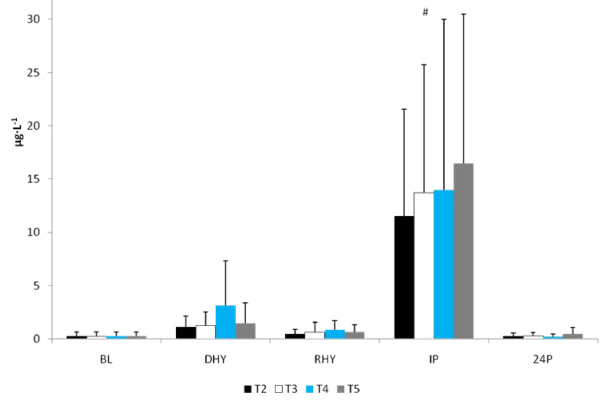
**Growth Hormone Response**. # = significant main effect for time versus BL, DHY RHY, and 24P

No significant differences from BL were seen in the testosterone response to the exercise and dehydration stress during any experimental trial (Figure 10). A significant main effect for time was seen in both the ACTH (p = 0.000) and cortisol (p = 0.000) response to the exercise and dehydration protocol (Figure 11 and 12, respectively). When collapsed across trials, significant elevations in cortisol and ACTH concentrations were seen at IP and 24P compared to BL, DHY and RHY. No other significant differences were noted and no between trial effects were observed. A significant main effect for time (p = 0.000) was seen in the growth hormone response. When collapsed across trials, growth hormone concentrations were significantly elevated at IP compared to all other time points (Figure 13). No other differences were observed. AUC analyses for testosterone, ACTH, cortisol and growth hormone did not result in any significant differences between trials. No significant difference from baseline concentrations (43.9 ± 18.7 IU) was seen in creatine kinase concentrations during any trial.

Plasma volumes decreased -5.45 ± 11.38% at DHY for all experimental trials, plasma volumes were decreased at RHY (-6.78 ± 11.27%) for all experimental trials and continued to decrease at IP (-21.44 ± 10.54%). However, the differences between trials were not significant. Blood variables were not corrected for plasma volume shifts due to the importance of molar exposure at the tissue receptor level.

## Discussion

The results of this study showed that when subjects are hypohydrated by 2.5% of their body mass and exercise to exhaustion, significant performance decrements occurred. However, when subjects ingested the AG supplement during the rehydration period (T4 and T5) the magnitude of performance decrement was significantly less compared to the dehydrated condition (T2). Water alone (T3) did not appear to significantly reduce the performance decrement. Despite significant performance improvements for both T4 and T5, glutamine concentrations were significantly elevated at only T5 for both RHY and IP compared to all other trials. As expected, the higher dose of AG produced a greater increase in plasma glutamine concentrations. The time course of glutamine appearance in plasma is similar to that reported by Klassen and colleagues [20]. In that study, a 20 g oral feeding (approximate to the high dose [T5] used in this study) resulted in a peak increase occurring at 49 ± 8 min (range 30 - 120 min) following dosing, which corresponded to the RHY and IP blood draws. Although dosing patterns of 0.1 g·kg·BM^-1 ^can increase plasma glutamine concentration by approximately 50% [21], the ability to increase plasma glutamine concentrations with doses lower than 0.1 g·kg·BM^-1 ^is not clear. Based on the present findings a dose of 0.05 g·kg·BM^-1 ^AG did not result in a significant elevation in plasma glutamine concentrations.

Despite the lack of any significant increase in plasma glutamine concentrations at T4, significant performance improvements were found for both T4 and T5. It is possible that in instances where plasma glutamine concentrations are normal, small bolus samples may be sufficient to offset mild hydration perturbations. The AG dipeptide has an important role in fluid and electrolyte uptake in the gut. AG appears to increase electrolyte and fluid uptake across the intestines by increasing ion transport through an enhanced signaling pathway within the intestinal mucosal cells [6,22]. Further, AG supplementation has also been demonstrated to enhance muscle glutamine uptake [23]. Although speculative, it is likely that an enhanced glutamine uptake by skeletal muscle will also result in a greater sodium uptake, which is supported by the reduced sodium concentrations at T4 - T5 compared to T2. The enhanced sodium uptake by skeletal muscle may have contributed to a reduction in fatigue by maintaining strength and efficiency of muscle contractility [24]. In addition, although plasma glucose concentrations were not different between trials, alanine is a gluconeogenic substrate and may have contributed to the delay in fatigue by sparing muscle glycogen [25,26].

ALD responses were significantly lower at RHY and IP for all trials, with no between trials differences observed. Although ALD is reported to respond in a graded manner to levels of hypohydration [27,28], the magnitude of hypohydration in this study was likely not sufficient to stimulate increased ALD production, and rehydration likely resulted in the significant decline of ALD across trials at RHY and IP. These findings agree with observations that ALD concentrations will decline when water or electrolyte drinks are provided during exercise [29]. The similarity in the ALD response found in this study may also be attributed to similar plasma volume changes observed between trials [29]. In addition, ALD is also stimulated by changes in BP [30]. In this present study, there was a significantly lower BP at IP during the T2 and T3 trials compared to T1. This most likely reflected a greater local fatigue resulting from the hydration perturbation contributing to the reduced time to exhaustion compared to T4 and T5 (an approximate 20 mmHg difference [p > 0.05] was found between post-exercise systolic BP at T2, T3 compared to T4 and T5). The significantly lower ALD responses at RHY and IP for all trials likely reflect the lack of a strong single stimulus (e.g. level of hypohydration) and represent the multitude of physiological factors that influence ALD secretion.

Our findings also indicated that AVP was significantly elevated from BL at all time points and that AVP concentrations at IP were significantly greater than DHY as well. However, the AG supplement was unable to alter the response of AVP to this mild dehydration and exercise protocol. The response to the exercise protocol was consistent with previous studies examining a similar exercise intensity [28]. Changes in AVP concentrations are dependent upon exercise intensity and changes in P_osm _and blood volume [31,32]; thus it is not surprising to see no significant differences between the trials in the AVP response considering that no between trial differences were noted in P_osm _or plasma volume changes. The mild dehydration and exercise protocol was unable to create any difference to the fluid regulatory response between the various trials. Previous studies examining the effect of hypohydration levels have typically examined body water deficits of greater magnitudes (~5%) and greater differentials than that used in this present study [28,29].

CRP is often used as a marker of inflammation and muscle damage [33-35]. Previous studies have shown that CRP will increase in response to prolonged physical activity such as triathlons [35] and marathons [33] but not during shorter duration exercise [34,36]. It is likely that the relatively short duration in time to exhaustion, despite the added mild dehydration stress did not cause a significant inflammatory response. Many studies use CK as a marker for muscle damage and have suggested that a rapid acute phase inflammatory response (reflected by an increase in CRP within 24 hours post-exercise during eccentric exercise in untrained individuals) can initiate delayed onset of muscle soreness and additional tissue necrosis occurring following 24 hours post-exercise is reflected by elevations in CK [37]. Although CRP concentrations observed in this study were significantly elevated from baseline levels, they were not different between DHY, RHY, IP and 24P suggesting that any changes may have been the result of plasma volume shifts and not due to an inflammatory response. This is supported by the response of CK during each trial (no change from baseline concentrations). Interestingly, the AG supplement was unable to provide any indication for an attenuation of the inflammatory or muscle damage response to the exercise protocol. This is likely due to the limited inflammatory response and lack of a clear indicator of muscle damage as measured by CK.

There was a significant elevation in serum concentrations of IL-6 at IP compared to BL, DHY and 24P and at RHY compared to BL and 24P. This response is consistent with previous studies that have shown significant elevations following prolonged endurance [33,35,38] and eccentric exercise [34]. IL-6 is produced in active skeletal tissue [39] and in the central nervous system [40]. Exercise is a potent stimulator of IL-6 production, with elevations greater than 100-fold reported [41]. It is thought that increases in IL-6 modulates CRP production in the liver [42] and operate synergistically to enhance the inflammatory response to exercise. The potential outcome from this inflammatory response is the risk for significant tissue damage and reduced recovery capability.

Several investigations have examined the ability of nutritional intervention to attenuate the post-exercise inflammatory response [43,44]. Carbohydrate ingestion [44] and a vitamin E and omega-3 fatty acid combination [43] have been successful in attenuating the IL-6 response to exercise. In contrast, glutamine supplementation has been shown to enhance plasma IL-6 production [38], while an AG dipepide has shown to have no effect on cytokine production in healthy individuals [45]. Hiscock and colleagues [38] suggested that the enhanced glutamine uptake by skeletal muscle would increase or maintain the production of IL-6. This hypothesis may be more consistent with the anti-inflammatory role suggested of IL-6 during exercise [46]. Increases in IL-6 concentrations have been consistently reported without corresponding muscle damage [46], and is supported by the results of this present study. The difference between this study and the results of Hiscock et al., [38] may be related to the length of exercise and the training experience of the subjects. In the present study the duration of exercise ranged from 5 - 47 minutes following the ~60 minute active dehydration protocol, in recreationally trained individuals, while the subjects in Hiscock's study were untrained and required to perform a 2-hr time trial using the same exercise intensity as employed in this study. However, those subjects were euhydrated and allowed to drink ad libitum. It is unlikely that dosing impacted these results, considering that the glutamine dose used in Hiscock's study (3.5 g) was similar to the low dosing trial (T4).

[MDA] were significantly elevated from baseline for all trials. This is not surprising considering that exercise is a potent stimulator of the formation of reactive oxygen species [47]. The results of this study are also consistent with previous research demonstrating elevated oxidative stress following a mild dehydration and exercise to exhaustion protocol [48]. However, in contrast to Paik and colleagues [48] rehydration with water, or water and the supplement, was unable to reduce the MDA response to the exercise protocol. It is likely that the differences between these two studies may reflect the methods used to achieve dehydration. Paik et al., [48] used passive means in the heat (sauna exposure) to achieve 3% hypohydration, while this present study used both a passive and active (exercise) dehydration protocol to achieve the 2.5% body weight loss. Although speculative, it is possible that differences between methods used for dehydration may have resulted in a different oxidative stress. The time used to achieve body weight loss, although performed at a lower intensity of exercise, resulted in significant elevations in MDA concentrations that were not altered by water or water and AG.

The anabolic and catabolic response to the study protocol did not differ among trials suggesting that the supplement was unable to provide any significant benefit regarding enhanced recovery from the exercise and hypohydration stress. It is also possible that these hormonal measures may not have been sensitive enough for assessing recovery from a moderate dehydration and endurance exercise protocol [49]. [TEST] did not significantly elevate from baseline levels following exercise despite a reduction in plasma volume. This is not surprising considering that subjects experienced only a moderate hypohydration stress and that time to exhaustion ranged from 13 - 18 minutes. Exercise of relatively short duration (i.e. 10-20 minutes) does not appear to increase [TEST] [50,51], even with a mild hydration perturbation in fit individuals [52].

The CORT response was consistent with previous studies that have shown that hydration levels do not influence [CORT] [52,53]. The post-exercise elevation in CORT was also consistent with the metabolic stress associated with moderate exercise and hypohydration [53,54]. Results of this study though were unable to show that CORT responses can differentiate between levels of hypohydration, which contrasts with observations made by Judelson et al., [55] and Maresh et al., [54]. However, the ability for hypohydration to modify the catabolic response to exercise appears to be more relevant when hypohydration reaches 5% or greater, or when exercise is performed at higher exercise intensities [54,55]. These findings also suggest that the pituitary-adrenal axis responds similarly to this exercise and hypohydration perturbation as ACTH responded in a similar pattern as CORT, with no influence from the AG supplementation.

GH secretion patterns have been shown to be quite responsive to changes in the acid-base balance of muscle [56]. Considering that no differences were noted in the La^- ^response between the trials, the GH response to the exercise and hypohydration stress appears to have responded in a normal manner. These results are also in agreement with Judelson et al., [55] but, contrast with Peyreigne and colleagues [57]. In this latter study, it was suggested that a hydration stress could blunt the GH response to exercise. Interestingly, there was an 18% and 20% greater GH response (p > 0.05) during T3 and T4 versus T2, respectively, while a 42% difference was found between T5 and T2 (p > 0.05). Although these responses were not significantly different, it does suggest an interesting trend that provided some support to previous results [57]. Whether the high dose glutamine ingestion played a role in this response is not clear. Previous investigations have suggested that glutamine concentrations can elevate the GH response at rest [58,59], but not exercise [58]. It appears that the most compelling stimulus for glutamine's role in stimulating GH release is during prolonged critical illness when plasma glutamine concentrations are below normal levels [60]. Thus, the high variability in the GH response in this study may be attributed to the normal glutamine concentrations at rest, however the largest gains in GH occurred during the trial (T5) that glutamine concentrations were significantly higher than T2 - T4.

In conclusion, the results of this study demonstrate that AG supplementation provides significant ergogenic benefits by increasing time to exhaustion during a mild hydration stress. This ergogenic effect was likely mediated by an enhanced fluid and electrolyte uptake. AG supplementation, irrespective of dosing, did not have any effect on immune, inflammatory or oxidative stress responses. Results also indicated that the AG supplement did not influence the pituitary-adrenal-testicular axis during this exercise and mild hypohydration perturbation.

## Competing interests

Kyowa Hakko USA (New York, NY) provided funding to The College of New Jersey for this project. All researchers involved independently collected, analyzed, and interpreted the results from this study and have no financial interests concerning the outcome of this investigation. Publication of these findings should not be viewed as endorsement by the investigator, The College of New Jersey or the editorial board of the Journal of International Society of Sports Nutrition.

## Authors' contributions

JRH was the primary investigator, obtained grant funds for project, designed study, supervised all study recruitment, data/specimen analysis, statistical analysis and manuscript preparation. NAR, JK, SLR, NK, AMG, MS, SA, and ADF oversaw all aspects of study including recruitment, data/specimen analysis, and manuscript preparation. BLB, LMY, LLH, BK and CMM were co-authors, assisting with data analysis. All authors have read and approved the final manuscript.
